# Development of Simultaneous Analysis of Thirteen Bioactive Compounds in So-Cheong-Ryong-Tang Using UPLC-DAD

**DOI:** 10.1155/2018/2875681

**Published:** 2018-05-02

**Authors:** Ji Hyun Jeong, Seon Yu Lee, Bo Na Kim, Guk Yeo Lee, Seong Ho Ham

**Affiliations:** National Development Institute of Korean Medicine, Udae land gil 288, Jangheung-gun, Jeollanam-do 59338, Republic of Korea

## Abstract

So-Cheong-Ryong-Tang, which is a standardized Korean medicine of the National Health Insurance, is a traditional prescription for the treatment of allergic rhinitis, bronchitis, and bronchial asthma. Simultaneous analysis and development of SCRT is essential for its stability, efficacy, and risk management. In this study, a simple, reliable, and accurate method using ultrahigh-performance liquid chromatography (UPLC) fingerprinting with a diode array detector (DAD) was developed for the simultaneous analysis. The chromatographic separation of the analytes was performed by an ACQUITY UPLC BEH C18 column (1.7 *μ*M, 2.1 × 100 mm, Waters) with a mobile phase of water containing 0.01% (v/v) phosphoric acid and acetonitrile containing 0.01% (v/v) phosphoric acid. The flow rate and detection wavelength were set at 0.4 mL/min and 215, 230, 254, and 280 nm. All calibration curves of the thirteen components showed good linearity (*R*^2^ > 0.999). The limit of detection and limit of quantification ranged 0.001–0.360 and 0.004–1.200 *µ*g/mL, respectively. The relative standard deviation (RSD) of intra- and interday was less than 2.60%, and the recoveries were within the range 76.08–103.79% with an RSD value of 0.03–1.50%. The results showed that the developed method was simple, reliable, accurate, sensitive, and precise for the quantification of bioactive components of SCRT.

## 1. Introduction

Traditional Korean medicines, because of their high effectiveness and low toxicity, have been used for thousands of years for the prevention and treatment of various kinds of human diseases. Various ingredients in these herbs cause the efficacy of traditional Korean medicines, which consist of many herbal combinations. Therefore, the consistency of the composition and proportion of the composition are the key to quality control in safety, efficacy, and risk management. In general, analyzing a single marker compound is simple and convenient, but it does not provide sufficient quantitative information on other components in Korean medicines. Thus, over the past decade, the chromatographic fingerprinting method has been considered one of the most important and acceptable approaches for the identification and quality evaluation of Korean medicines [1, 2]. So-Cheong-Ryong-Tang, standardized as a Korean medicine of the National Health Insurance, is a traditional prescription for the treatment of allergic rhinitis, bronchitis, and bronchial asthma [3]. Recently, SCRT was reported to show therapeutic effects in *in vivo* experiments on the respiratory system for allergic rhinitis and asthma [3–9]. So-Cheong-Ryong-Tang (SCRT, Xiao-Qing-Long-Tang in Chinese, Sho-seiryu-to in Japanese) is composed of eight herbal preparations (*Ephedrae herba, Paeoniae radix, Glycyrrhizae radix*, *Zingiberis rhizoma*, *Cinnamomi ramulus*, *Schisandrae fructus*, *Pinelliae rhizoma*, and *Asiasari radix*) [4–6, 8].

To optimize the quality control of SCRT, thirteen bioactive compounds from eight herbal preparations were chosen. Among the 13 standard compounds, ephedrine and catechin were found in *Ephedrae herba*, which is known for its efficacy as a sympathomimetic and its antiobesity effects [10]. Albiflorin, paeoniflorin, benzoic acid, PGG, and methyl gallate are the major constituents of *Paeoniae radix*, which has anti-inflammatory, analgesic, antispasmodic, liver protection, and immune regulatory functions [11, 12]. Liquiritin, Liquiritin apioside, and Glycyrrhizin from *Glycyrrhizae Radix* were used, which is an effective detoxifying agent, presenting neuroprotective effect, antiviral activity, and anti-inflammatory, antitumor, and antibiosis effects [13]. 6-Shogaol from *Zingiberis Rhizoma* was found to have various pharmacological activities, including antioxidative, antitumorigenic, and immunomodulatory effects, and is an effective antimicrobial and antiviral agent [14]. *Cinnamomi ramulus*, which includes cinnamic acid, has been found to be able to effectively attenuate influenza virus, inflammations, human platelet aggregation, and arachidonic acid metabolism [15] and is known for its antimicrobial activity against [16]. The major constituent of *Schisandrae fructus*, which includes schisandrin, was found to have liver protective, hypoglycemic, antioxidant, antiaging, immune regulatory, antitumor, and bactericidal effects and plays a role in regulating the central nervous system [17]. *Pinelliae rhizoma* has antitussive, antiemetic, glandular secretion-inhibiting, and antitumor effects [18]. *Asiasari radix* has been used as an analgesic, antitussive, or antiallergic agent [19].

Several studies of these compounds have been developed for qualitative and quantitative analyses using high-performance liquid chromatography-diode array detector (HPLC-DAD) and mass spectrometry (HPLC-ESI-MS) [4, 5, 9]. However, these methods cannot offer simultaneous analysis of the multiple bioactive compounds in SCRT. Although an HPLC method for simultaneous determination of the four marker constituents of SCRT has been developed, there are limitations to the quantitative and qualitative analyses of many compounds in SCRT. Therefore, methods for simultaneously detecting these biomarkers in SCRT are essential to ensure efficient quality control and pharmaceutical evaluation. In this study, it was necessary to find more accurate, efficient, and stable solvent extraction conditions prior to simultaneous analysis. We performed simultaneous determination of the thirteen marker compounds.

## 2. Experimental Materials and Reagents

### 2.1. Chemicals and Reagents

**Figure 1:**
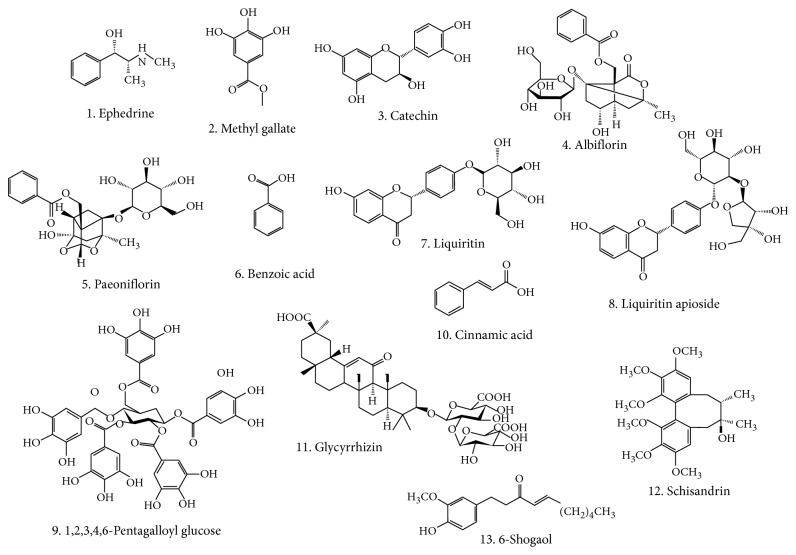
Chemical structures of the 13 marker compounds.

Ephedrine, methyl gallate, catechin, albiflorin, paeoniflorin, benzoic acid, liquiritin, liquiritin apioside, 1,2,3,4,6-pentagalloyl glucose (PGG), cinnamic acid, glycyrrhizin, schisandrin, and 6-shogaol were purchased from Sigma-Aldrich Co. (St. Louis, MO, USA). The purity of all standards was >97%.  Figure 1 shows the chemical structures of the thirteen bioactive compounds. HPLC-grade acetonitrile and methanol were purchased from J. T. Baker Inc. (Phillipsburg, NJ, USA). Deionized water was prepared using an ultrapure water production apparatus (Human Corporation, Seoul, Korea). SCRT medicines were purchased from Kyoungbang Medicinal Herbs (Incheon, Korea).

### 2.2. Preparation of Standard and Sample Solutions

Standard stock solutions of the thirteen bioactive standards—ephedrine, methyl gallate, catechin, albiflorin, paeoniflorin, benzoic acid, liquiritin, liquiritin apioside, 1,2,3,4,6-pentagalloyl glucose (PGG), cinnamic acid, glycyrrhizin, schisandrin, and 6-shogaol—were prepared by accurately weighing appropriate amounts of reference compounds and dissolving in methanol. The thirteen bioactive standards were mixed in stock solutions and then diluted serially to seven concentrations for the construction of calibration curves. All the solutions were stored at 4°C.

### 2.3. Extraction Method

The herbal medicine prepared by water extraction contains a water-soluble component and some lipoid-soluble substances, and most of the high molecular weight polymers are contained in a suspended state. It is necessary to estimate and optimize the extraction conditions for the 13 marker components in the SCRT sample. In this study, the liquid extraction method was selected, and aqueous methanol (20, 50, and 80%) was tried and examined as the extraction solvent to evaluate the optimal extraction solvent. Second, the volume (50 and 100 mL) of the extraction solvent was investigated. Finally, extraction methods using ultrasonic or reflux were investigated [20–22].

### 2.4. Preparation of Sample Solution

The SCRT powder (2.4 g, 1 dose) was weighed precisely and extracted with 80% methanol-water (v/v) solution in an ultrasonic water bath for 10 min at room temperature. Then, the samples were refluxed twice at 80°C for 30 min, followed by filtration and making up to volume in a volumetric flask. The samples were centrifuged (4,000 rpm, 10 min, 18°C), and the supernatant was filtered with a 0.2 *µ*m membrane filter, prior to injection. All working solutions and sample solutions were stored at 4°C before use.

### 2.5. Chromatographic Conditions by UPLC-DAD

The simultaneous determination of thirteen bioactive compounds in SCRT was performed on the UPLC-DAD system equipped with a pump, an autosampler, and a photodiode array detector, and the amount of data were calculated using Empower software. Chromatographic separation was carried out using an ACQUITY UPLC BEH C18 column (1.7 *μ*m, 2.1 × 100 mm, Waters), and the column temperature was kept at 40°C. The mobile phase consisted of (A) water (0.01% phosphoric acid, v/v) and (B) acetonitrile (0.01% phosphoric acid, v/v). The gradient solvent was optimized and performed as 98% A (0-1 min), 98–84% A (1–14 min), 84–60% A (14–21 min), 60–20% A (21–28 min), 20% A (28-29 min), and 20–98% A (29-29.5 min), at a flow rate of 0.4 mL/min. The detection wavelengths for analytes were set at 215, 230, 254, and 280 nm, and the injection volume of each sample was 2 *μ*L.

### 2.6. Method Validation

The method was validated for linearity, limit of detection (LOD), limit of quantification (LOQ), specificity, precision (interday, intraday, and repeatability), and accuracy (recovery), following the guideline on Bioanalytical Method Validation [23–26].

#### 2.6.1. Linearity, Limits of Detection (LODs), and Limits of Quantification (LOQs)

The standard solutions of the 13 compounds were prepared by serially diluting the stock solution to appropriate concentrations for plotting the calibration curves [28, 29]. These solutions of the 13 compounds were analyzed in triplicate [28]. The calibration standard curve was plotted with the peak area (*y*-axis) versus concentration (*x*-axis) for each analyte in that range [30]. All calibration curves were required to have a correlation value of at least 0.995. The limit of detection (LOD) and limit of quantification (LOQ) were determined on the basis of signal-to-noise ratio (S/N):

(1) $$\begin{array}{c} \begin{array}{l} \text{LOD}=\frac{\text{amount}*3.3}{\left(S/N\right)},\\ \mathrm{LOQ}=\frac{\text{amount}*10}{\left(S/N\right)}. \end{array} \end{array}$$

#### 2.6.2. Precision, Accuracy, and Recovery

The precision of the method was evaluated by both intra- and interday tests. Three different concentrations of each biomarker in five replicates on the same day (intraday) and on three consecutive days (interday) were prepared to verify the precision and accuracy of the analytical method [30]. The precision was expressed as relative standard deviation (RSD, %); a value of RSD within ±15% is generally considered acceptable [28]. The accuracy of the assay is the closeness of the observed concentration to the nominal concentration [31]. The recoveries of analytes were determined by adding different concentrations of the 13 marker components into the SCRT sample solution (2.4 mg/mL). Recovery (%) was calculated with the following equation:

(2) $$\begin{array}{c} \text{Recovery}\left(\%\right)=\frac{\left(\text{amount}\;\;\mathrm{found}-\text{original}\;\;\mathrm{amount}\right)}{\text{amount}\;\;\mathrm{spiked}}\times 100\%. \end{array}$$

## 3. Results and Discussion

### 3.1. Optimization of Chromatographic Condition

**Figure 2:**
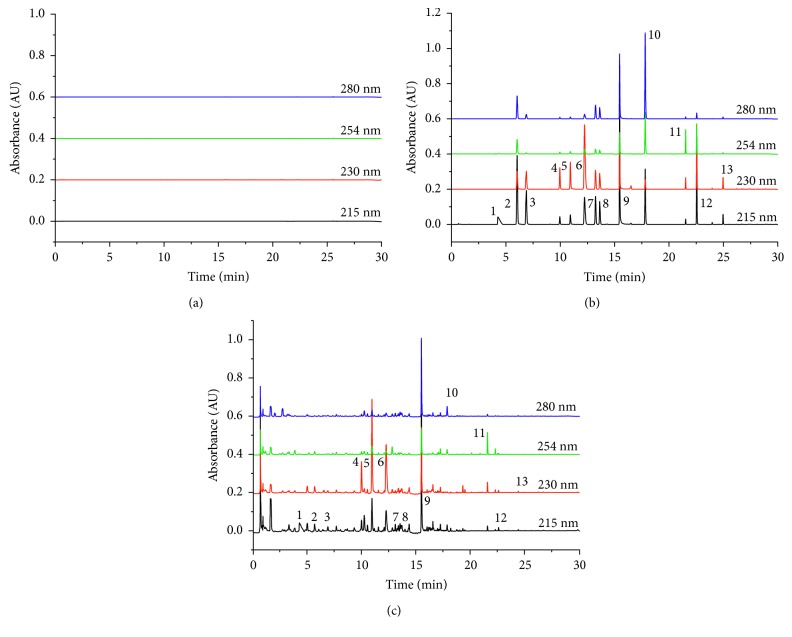
UPLC chromatograms of the (a) blank solvent, (b) standard mixture, and (c) SCRT extract.

A chromatogram of SCRT was obtained using an UPLC-PDA [4].  Figure 2 shows typical chromatograms corresponding to the mixed standard and SCRT. To obtain accurate, valid, and optimal separation, the UPLC conditions were investigated with regard to column, mobile phase (water-acetonitrile with different modifiers including acetic acid, formic acid, and phosphoric acid), detection wavelength (215, 230, 254, and 280 nm), and mobile-phase flow rate (0.5, 0.4, 0.3, and 0.2 mL/min) [32] The best UPLC conditions were obtained from the ACQUITY UPLC BEH C18 column, which had better resolution than the others. The gradient solvent system consisted of 0.01% phosphoric acid in water (A) and 0.01% phosphoric acid in acetonitrile (B), at a column temperature of 40°C, with a flow rate of 0.4 mL/min. Four detection wavelengths (215, 230, 254, and 280 nm) were finally selected to achieve the goal of high detection sensitivity and small interference because the maximum absorption of the 13 reference compounds was different.

### 3.2. Optimization of the Sample Extraction Protocol

**Figure 3:**
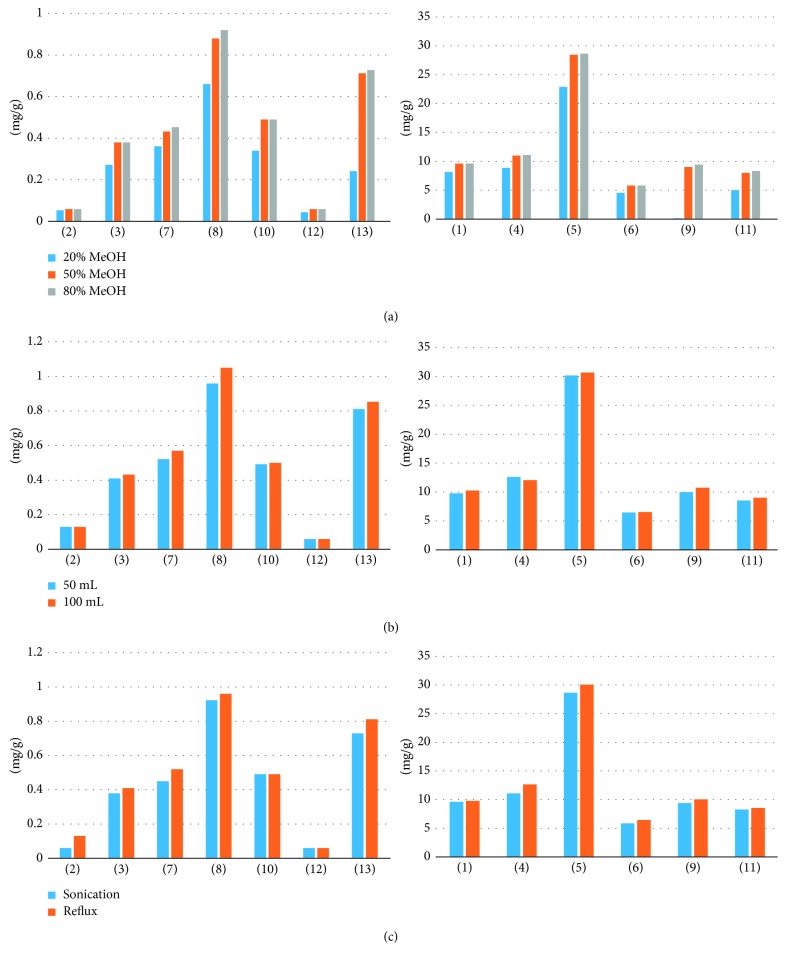
Efficiencies of the extraction for the nine compounds in SCRT using different (a) extraction solvents, (b) volumes of the extraction solvent, and (c) extraction methods.

The extraction conditions, for example, extraction solvent, method, and volume, can easily influence the efficiency of the extraction. In this paper, aqueous methanol (20, 50, and 80%) was examined as the extraction solvent for SCRT by ultrasonication for 30 min [33].  Figure 3(a) shows the results, which indicate that 80% methanol was the best extraction solvent. Second, the volume of solvent (50 and 100 mL) was investigated, and Figure 3(b) shows the results. The volume of the extraction solvent was 100 mL. The extraction efficiency was better at 100 mL volume in low-content compounds. Finally, Figure 3(c) shows the results of the extraction method using ultrasound and reflux, which indicate that the extraction method using reflux showed high efficiency. In the end, the suitable extraction conditions were as follows: the samples were extracted by reflux with 80% methanol in a volume of 100 mL.

### 3.3. Method Validation

#### 3.3.1. Specificity

**Figure 4:**
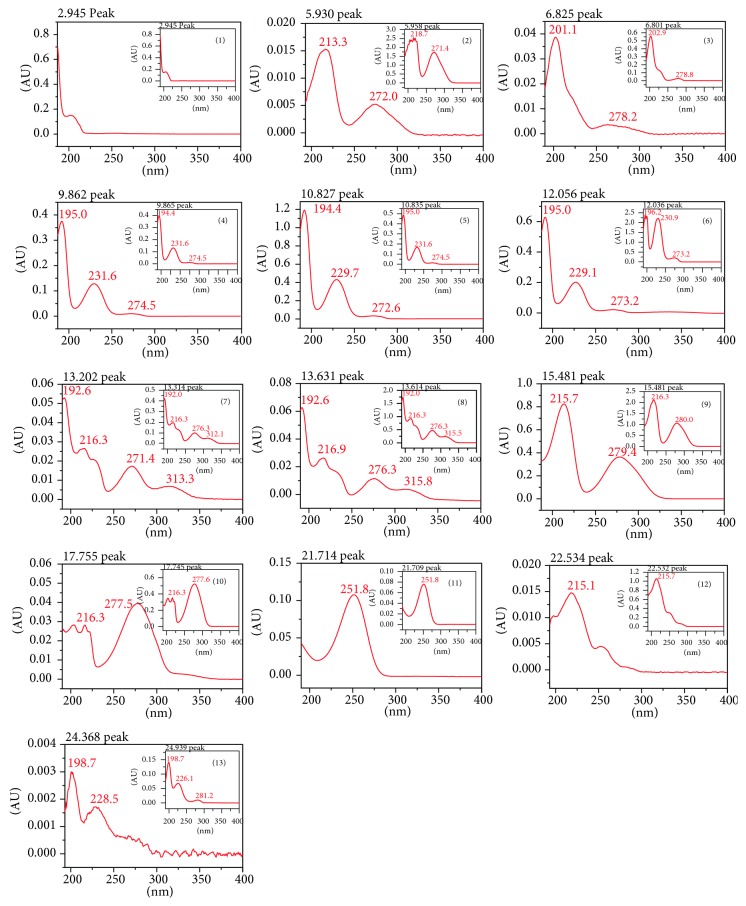
UV spectra of the thirteen marker compounds in SCRT corresponding to the standard solution (inset).

The specificity was determined by comparing the peak purity of the 13 markers with the extracted samples and the standard.  Figure 4 shows the UV spectra of individual marker compounds, which confirm that the peaks are pure and there is no interference from the impurities [34].

#### 3.3.2. Linearity, LOD, and LOQ

Table 1 summarizes the calibration curves, LOQ, and LOD of the thirteen analytes. The linearity of the developed method was assessed using seven concentrations: ephedrine, albiflorin, paeoniflorin, benzoic acid, PGG, glycyrrhizin (0.1∼100 *μ*g/mL), methyl gallate, catechin, liquiritin, liquiritin apioside, cinnamic acid (0.05∼50 *μ*g/mL), and schisandrin (0.02∼20 *μ*g/mL) with correlation coefficients *R*^2^ ≥ 0.999. The values of LOD and LOQ were in the ranges of 0.001–0.360 *µ*g/mL and 0.004–1.200 *µ*g/mL, respectively. The results showed that the calibration curves were within the adequate range and exhibited good sensitivity for the analysis of the thirteen bioactive components [28].

#### 3.3.3. Precision, Accuracy, and Recovery

Table 2 shows the results of the intra- and interday precision tests. The values of RSD (%) for intra- and interday tests were within the ranges of 0.01–2.51% and 0.02–2.60%, with accuracy from 96.82 to 102.36% and from 97.67 to 102.62%, respectively [2]. The results indicated that the accuracy and precision of the proposed method were accurate and reliable for determination of the thirteen compounds in the sample of SCRT [28]. The average recovery (%) of the 13 marker compounds shows the accuracy of this analytical method. The recovery ranged from 76.08 to 103.79%, and the values of RSD were in the range 0.03–1.50%.  Table 3 summarizes these results; they indicate that the proposed method enables highly accurate simultaneous analysis of the thirteen compounds [35].

## 4. Discussion

Standardization and analysis of the marker compounds in herbal medicines are necessary for safety, efficacy, and risk management. A simple, reliable, and accurate method using ultrahigh-performance liquid chromatography (UPLC) fingerprinting with a diode array detector (DAD) was developed for the simultaneous qualitative and quantitative analyses of the thirteen biomarkers: ephedrine, methyl gallate, catechin, albiflorin, paeoniflorin, benzoic acid, liquiritin, liquiritin apioside, 1,2,3,4,6-pentagalloyl glucose (PGG), cinnamic acid, glycyrrhizin, schisandrin, and 6-shogaol in SCRT.

To optimize the quality control of SCRT, thirteen bioactive compounds from eight herbal preparations (*Ephedrae herba, Paeoniae radix, Glycyrrhizae radix*, *Zingiberis rhizoma*, *Cinnamomi ramulus*, *Schisandrae fructus*, *Pinelliae rhizoma*, and *Asiasari radix*) were chosen.

However, *Pinelliae rhizoma* and *Asiasari radix* were not detected in the SCRT extract sample. Because the contents of bioactive compounds in herbal medicines can differ as a function of the collection period, region, species, and preparation method, or the medicine contains a concentration lower than the LOD value, further studies should be required to perform quantification and qualification analyses, using a standard addition method or ultrahigh-performance liquid chromatography-tandem mass spectrometry (UPLC-MS/MS).

The results show that all biomarker components were detected (Figure 3), and identified based on the UV absorbance spectra (Figure 4) and retention times, by comparison with standard compounds. The UPLC-DAD method was validated, and the results showed good linearity, LOD, LOQ, precision, and accuracy, with RSD < 2.51%. Furthermore, the method did not interfere with other chemical constituents in SCRT. The results show that our method is accurate and reliable for quantification and qualification of the bioactive components of SCRT.

## 5. Conclusion

The developed UPLC-DAD fingerprinting method for the simultaneous determination of thirteen biomarkers in SCRT, which include ephedrine, methyl gallate, catechin, albiflorin, paeoniflorin, benzoic acid, liquiritin, liquiritin apioside, PGG, cinnamic acid, glycyrrhizin, schisandrin, and 6-shogaol, proved an efficient tool for quality control and pharmaceutical evaluation. The results demonstrated that the developed UPLC-DAD method is simple, reliable, accurate, sensitive, and precise for quantification and qualification analyses of SCRT.

## Figures and Tables

**Table 1:** Regression data, LODs, and LOQs for the marker compounds analyzed by UPLC.

	Compound	Regression equation	*R* ^2^	LOQ (*µ*g/mL)	LOD (*µ*g/mL)
1	Ephedrine	*y* = 6,569.4*x* − 2,539.7	0.9996	0.070	0.233
2	Methyl gallate	*y* = 49,631*x* − 14,901	0.9995	0.007	0.026
3	Catechin	*y* = 28,138*x* − 4,098.4	0.9992	0.008	0.029
4	Albiflorin	*y* = 6,199.8*x* − 2,341.7	0.9996	0.040	0.134
5	Paeoniflorin	*y* = 8,506.1*x* − 1,169.5	0.9994	0.018	0.062
6	Benzoic acid	*y* = 30,844*x* − 11,922	0.9995	0.021	0.070
7	Liquiritin	*y* = 19,772*x* − 3,660	0.9995	0.017	0.056
8	Liquiritin apioside	*y* = 15,249*x* − 1,630.9	0.9996	0.145	0.483
9	1,2,3,4,6-Pentagalloyl glucose	*y* = 29,863*x* − 17,029	0.9994	0.009	0.031
10	Cinnamic acid	*y* = 25,479*x* − 5,716.6	0.9994	0.008	0.027
11	Glycyrrhizin	*y* = 3,546.4*x* − 422.55	0.9994	0.073	0.244
12	Schisandrin	*y* = 86,584*x* − 4,328.7	0.9995	0.001	0.004
13	6-Shogaol	*y* = 1,025.4*x* + 1,176.6	0.9988	0.360	1.200

*R* ^2^ = correlation coefficient; *y* = peak area; *x* = sample concentration (*µ*g/mL).

**Table 2:** Precision (intra- and interday) and accuracy of the thirteen analytes.

Analyte concentration (*µ*g/mL)	Intraday (*n*=5)			Interday (*n*=3)		
	Measured amount (mean ± SD, Dg/mL)	RSD (%)	Accuracy (%)	Measured amount (mean ± SD, Dg/mL)	RSD (%)	Accuracy (%)
Ephedrine						
25	5.03 ± 0.02	0.43	100.59	4.97 ± 0.04	0.82	99.30
50	25.59 ± 0.01	0.03	102.36	25.33 ± 0.23	0.90	101.31
100	100.46 ± 0.08	0.08	100.46	100.46 ± 0.31	0.31	100.46

Methyl gallate						
2.5	2.48 ± 0.04	1.49	99.36	2.51 ± 0.01	0.35	100.57
12.5	12.29 ± 0.00	0.03	98.34	12.44 ± 0.12	1.00	99.48
50	49.14 ± 0.31	0.63	98.27	50.03 ± 1.00	1.99	100.06

Catechin						
2.5	2.53 ± 0.00	0.12	101.09	2.52 ± 0.02	0.75	100.65
12.5	12.50 ± 0.00	0.02	100.00	12.50 ± 0.00	0.02	100.02
50	49.69 ± 0.21	0.43	99.38	50.14 ± 0.48	0.96	100.28

Albiflorin						
5	5.02 ± 0.03	0.61	100.36	5.12 ± 0.07	1.42	102.42
25	25.05 ± 0.04	0.15	100.20	25.12 ± 0.11	0.43	100.47
100	98.68 ± 0.16	0.16	98.68	99.78 ± 0.80	0.80	99.78

Paeoniflorin						
5	5.05 ± 0.10	1.94	101.10	5.13 ± 0.04	0.73	102.62
25	25.24 ± 0.08	0.30	100.96	25.11 ± 0.27	1.07	100.44
100	101.46 ± 1.96	0.93	101.46	100.46 ± 0.15	0.15	100.46

Benzoic acid						
1	1.01 ± 0.01	1.35	100.92	1.01 ± 0.02	1.61	101.28
5	5.02 ± 0.02	0.39	100.31	4.99 ± 0.04	0.80	99.80
100	96.82 ± 2.43	2.51	96.82	100.59 ± 0.28	0.28	100.59

Liquiritin						
2.5	2.49 ± 0.01	0.57	99.64	2.44 ± 0.04	1.64	97.67
12.5	12.14 ± 0.01	0.05	97.10	12.37 ± 0.21	1.66	98.99
50	50.20 ± 0.29	0.58	100.39	50.17 ± 0.48	0.96	100.35

Liquiritin apioside						
2.5	2.53 ± 0.03	1.08	101.11	2.51 ± 0.00	0.16	100.45
12.5	12.56 ± 0.00	0.02	100.48	12.51 ± 0.04	0.34	100.07
50	49.91 ± 0.36	0.72	99.81	49.95 ± 0.09	0.17	99.90

1,2,3,4,6-Pentagalloyl glucose						
5	4.94 ± 0.07	1.45	98.73	5.05 ± 0.03	0.68	101.00
25	25.26 ± 0.02	0.08	101.04	25.15 ± 0.10	0.38	100.60
100	99.89 ± 0.45	0.45	99.89	100.27 ± 0.37	0.37	100.27

Cinnamic acid						
0.5	0.50 ± 0.00	0.38	100.16	0.50 ± 0.00	0.33	99.45
12.5	12.50 ± 0.00	0.02	99.96	12.51 ± 0.02	0.13	100.06
50	50.03 ± 0.01	0.02	100.06	50.15 ± 0.19	0.38	100.31

Glycyrrhizin						
5	5.03 ± 0.02	0.30	100.62	4.99 ± 0.11	2.27	99.84
25	25.04 ± 0.11	0.42	100.14	25.09 ± 0.01	0.05	100.35
100	100.63 ± 0.30	0.29	100.63	100.65 ± 0.17	0.17	100.65

Schisandrin						
1	1.00 ± 0.00	0.11	100.03	1.00 ± 0.00	0.40	100.40
5	5.09 ± 0.00	0.01	101.83	5.04 ± 0.05	0.90	100.89
20	19.33 ± 0.42	2.17	96.64	20.12 ± 0.18	0.87	100.60

6-Shogaol						
2	2.04 ± 0.04	2.00	101.93	2.05 ± 0.05	2.60	102.50
50	50.36 ± 0.04	0.09	100.71	50.27 ± 0.18	0.35	100.54
200	197.59 ± 0.09	0.05	98.79	199.67 ± 1.72	0.86	99.83

SD = standard deviation; RSD (%) = (SD/mean) × 100; accuracy (%) = (*C*_obs_/*C*_nom_) × 100.

**Table 3:** Determination of recoveries of the 13 compounds (1–13) in SCRT.

Compounds	Spiked amount	Measured amount	Recovery (%)	RSD (%)
Ephedrine	25	25.20 ± 0.19	100.78	0.73
	50	51.86 ± 0.78	103.72	1.50
	100	100.23 ± 0.59	100.23	0.59

Methyl gallate	5	4.47 ± 0.01	89.33	0.30
	12.5	10.94 ± 0.04	87.53	0.33
	50	45.63 ± 0.03	91.26	0.07

Catechin	5	4.98 ± 0.02	99.53	0.40
	12.5	11.42 ± 0.09	91.39	0.76
	50	45.53 ± 0.30	91.05	0.65

Albiflorin	5	5.15 ± 0.02	103.09	0.36
	25	25.90 ± 0.11	103.59	0.42
	100	98.23 ± 1.06	98.23	1.08

Paeoniflorin	10	10.05 ± 0.02	100.54	0.21
	25	24.64 ± 0.08	98.55	0.34
	100	100.97 ± 0.35	100.97	0.35

Benzoic acid	25	24.43 ± 0.09	97.70	0.39
	50	47.29 ± 0.17	94.59	0.37
	100	97.28 ± 0.10	97.28	0.10

Liquiritin	5	5.05 ± 0.05	100.93	0.91
	12.5	12.54 ± 0.12	100.31	0.97
	50	42.98 ± 0.22	85.96	0.52

Liquiritin apioside	5	4.94 ± 0.04	98.79	0.79
	12.5	12.55 ± 0.03	100.43	0.27
	50	48.99 ± 0.45	97.98	0.93

1,2,3,4,6-Pentagalloyl glucose	10	10.00 ± 0.13	99.98	1.30
	50	46.89 ± 0.25	93.77	0.53
	100	99.17 ± 0.56	99.17	0.56

Cinnamic acid	12.5	12.61 ± 0.06	100.88	0.49
	25	24.70 ± 0.02	98.81	0.09
	50	52.28 ± 0.22	104.57	0.42

Glycyrrhizin	25	24.13 ± 0.16	96.54	0.66
	50	50.36 ± 0.14	100.72	0.27
	100	102.79 ± 0.52	102.79	0.50

Schisandrin	1	0.98 ± 0.01	97.78	0.87
	5	4.65 ± 0.04	92.93	0.77
	20	17.98 ± 0.03	89.90	0.18

6-Shogaol	20	15.22 ± 0.02	76.08	0.14
	100	85.23 ± 0.05	85.23	0.06
	200	177.66 ± 0.05	88.83	0.03
